# Joint attention supports working memory when gaze cues are reliable and task-relevant

**DOI:** 10.3758/s13414-025-03163-x

**Published:** 2025-12-02

**Authors:** Caterina Foglino, Agnieszka Wykowska

**Affiliations:** Social Cognition in Human-Robot Interaction, Italian Institute of Technology, Via Enrico Melen 83, 16152 Genova, Italy

**Keywords:** Gaze cueing, Visual working memory, Top-down control, Joint attention

## Abstract

This study investigated whether attentional orienting in response to gaze cues enhances visual working memory (WM) automatically, or whether engagement of top-down processes is necessary for such effects to emerge. Building on an existing gaze-cueing paradigm, we tested whether joint attention supports WM under two conditions. In Experiment 1, participants viewed centrally presented static images of human faces displaying directional gaze cues without any instruction to use gaze direction, and gaze validity was set at 50%, making the cue spatially uninformative of stimuli location. Following the cue, a memory array was presented, followed by a retention interval and a single-probe recall. Participants had to indicate whether the probe had appeared in the initial memory set. No WM advantage was found for validly cued items. In Experiment 2, we increased cue validity to 75% and explicitly informed participants that gaze direction was highly predictive of stimuli location. Under this condition, which presumably elicited higher engagement of top-down processes, valid gaze cues significantly enhanced WM performance relative to invalid cues. Interestingly, as cognitive load increased, the limited capacity of WM slightly constrained the extent to which this strategic orienting could translate into improved memory sensitivity. These results highlight the interplay between cue reliability, attentional control, and WM capacity in determining the efficacy of gaze cues. Our findings clarify the conditions under which joint attention facilitates WM and contribute to a growing literature showing that social attention effects on higher-level cognition are context-sensitive and cognitively mediated.

## Introduction

Joint attention (JA) ‒ the shared focus of two individuals on an object or event ‒ is a fundamental mechanism supporting human communication and social cognition (Frischen et al., 2007; Mundy, 2018). A key behavior that enables JA is the orienting of one’s attention in response to another’s gaze direction. This mechanism of gaze-mediated orienting is widely studied using the gaze-cueing paradigm. In this task, a centrally presented face initially gazes forward and then shifts its gaze to the left or right (Friesen & Kingstone, 1998). Shortly afterward, a target appears at either the gazed-at location (valid trial) or the opposite location (invalid trial). Participants typically respond faster and more accurately to validly cued targets, demonstrating the powerful influence of gaze direction on attentional orienting. Notably, this effect occurs when gaze direction is non-predictive (Friesen & Kingstone, 1998; Frischen et al., 2007) and even counter-predictive of target location (Driver et al., 1999; Friesen et al., 2004), suggesting that reflexive, bottom-up mechanisms are sufficient to trigger attentional shifts. These findings support the idea that gaze serves as a biologically and socially relevant attentional cue, capable of rapidly redirecting attention (Emery, 2000; McKay et al., 2021).

However, subsequent research has challenged the view that gaze cueing is to a large extent automatic. Several studies suggest that gaze-driven orienting is flexible and context-sensitive, modulated by top-down factors such as the perceived intentionality of the cue agent (Wiese et al., 2012), the agent’s visual perspective (Nuku & Bekkering, 2008; Teufel et al., 2010), or beliefs about the agent’s agency and cue informativeness (Wiese et al., 2014; Wykowska et al., 2014). Characteristics such as familiarity, trustworthiness, and social status of the gaze agent modulate cueing effects (Dalmaso et al., 2020), highlighting how social context and interpersonal expectations shape gaze-guided attention. Yet, with other studies finding limited evidence for such top-down modulation (Cole et al., 2015), the extent to which gaze-based orienting is shaped by higher-level processes remains under debate.

The influence of this gaze-mediated orienting extends beyond simple target detection, with a growing body of research showing it can significantly modulate memory performance. Behavioral and neurophysiological evidence demonstrates that eye gaze cues can modulate memory across encoding, maintenance, and retrieval stages (Yin, 2022). For example, Dodd et al. (2012) found that participants in their study were more likely to recognize items that had previously been looked at by a centrally presented face, during encoding, and Gregory and Jackson (2017) demonstrated that WM performance improves for items jointly attended with a centrally presented face cue. Interestingly, this effect was specific to gaze cues and did not generalize to nonsocial directional cues like arrows, highlighting the social specificity of the mechanism. Similarly, Nie et al. (2018) found that gaze cues selectively increased the likelihood that gaze-congruent items would be retained in visual WM. suggesting a reflexive prioritization mechanism at the encoding stage.

Other findings point to the importance of social interpretation and task specificity in the context of JA and memory too. For example, Gregory and Jackson (2019) found that the WM advantage from gaze cues disappears when a physical barrier blocks the cue agent’s view, suggesting that the observer’s belief in the agent’s ability to see ‒ i.e., perspective-taking ‒ modulates the effect. Building on this, Gregory et al. (2022) showed that valid gaze cues elicit neural markers of attentional prioritization and cognitive control in a spatial WM task in virtual reality, indicating that when gaze cues are task-relevant (e.g., when cues have high cue validity and participants are explicitly informed of their informativeness), they engage top-down mechanisms to support memory encoding. These findings suggest that gaze-driven memory benefits may rely on strategic use of gaze information rather than purely reflexive orienting. Despite this growing literature, it remains unclear whether reflexive orienting to gaze is sufficient to boost memory, or whether strategic use of gaze information ‒ driven by task demands or expectations ‒ is required.

To address the question of the degree of involvement of top-down processes in gaze cueing enhancing memory performance, we adapted the gaze-cueing paradigm developed by Gregory and Jackson (2017) and manipulated the degree of top-down engagement in two WM experiments. In Experiment 1, we removed all instructions that might explicitly direct participants to attend to the centrally presented gaze cue, and set the cue validity at 50%, making it non-predictive of stimuli location. This condition was designed to assess whether gaze-triggered attention alone could still facilitate visual WM. In Experiment 2, we increased the cue validity to 75% and explicitly instructed participants that gaze direction often indicated the location of the stimuli to memorize. This manipulation aimed to encourage participants to strategically use gaze information.

## Method

### Participants

An a priori power analysis using G*Power (Faul et al., 2007) was based on the effect size reported in Gregory and Jackson (2017; Experiment 1: Cohen’s d = 0.358 for valid vs. invalid conditions). This indicated that approximately 30 participants would be sufficient to detect a medium effect (α =.05, power =.80). Consequently, 30 participants performed Experiment 1 (mean age = 26.73, SD = 9.47 years; female = 24; left-handed = 3), and 30 participants took part in Experiment 2 (mean age = 32.23, SD = 10.93 years; female = 16; left-handed = 4).

All participants reported normal or corrected-to-normal vision, including no color vision deficiency. All participants provided written informed consent and were compensated with a 10€ honorarium for taking part in the study. Both Experiment 1 and Experiment 2 were carried out in line with the ethical principles outlined in the 2013 Declaration of Helsinki and received approval from the local ethics committee (Comitato Etico Regione Liguria).

### Apparatus and stimuli

All participants performed the task while seated at a desk, with stimuli presented on a monitor positioned 60 cm away from them. In Experiment 1, stimuli were presented on a Dell P2217H monitor (1,920 × 1,080 pixels, 21.5-in.). In Experiment 2 stimuli were presented on a Dell U2412M (1,920 × 1,200 pixels, 24-in.). Both tasks were presented using PsychoPy® version 2022.2.4. Ten colored squares (black, blue, brown, green, orange, pink, purple, red, turquoise, and yellow) served as the stimuli to memorize in both Experiment 1 and Experiment 2. Each square subtended 0.8° × 0.8° of visual angle. The central gaze cues consisted of human faces presented in grayscale. The gaze cue subtended 3.2° × 4.5° of visual angle. Three images were used: one with a direct gaze, one with a leftward gaze, and one with a rightward gaze. The human central cues used in this study were identical to those employed in Gregory and Jackson (2017) and were provided by these authors upon request. To ensure consistency in facial structure across gaze directions, the eye regions from the leftward and rightward gaze images were cropped and overlaid onto the direct gaze image, such that only the direction of the eyes varied. One female face and one male face were selected from the original set of stimuli.

### Design and procedure

Two levels of cue validity were tested: valid and invalid. The gender of the human cues was balanced within each block, with half of the trials featuring a female face and the other half a male face (pseudo-randomized within the block). WM load (four, six, eight items) was manipulated so that each memory load appeared in one-third of the trials per block. Target color presence (present or absent) and color set location (left or right) were pseudorandomized so that each condition occurred on 50% of the trials within each block. Color set combinations and target color were randomly selected on each trial. The experiment consisted of six blocks of 72 trials each, with breaks allowed between blocks. Each participant performed ten practice trials before starting the experiment. Response keys (“q” and “p”) were counterbalanced across participants for indicating target presence. The whole experimental procedure lasted approximately 1 h.

**Fig. 1:**
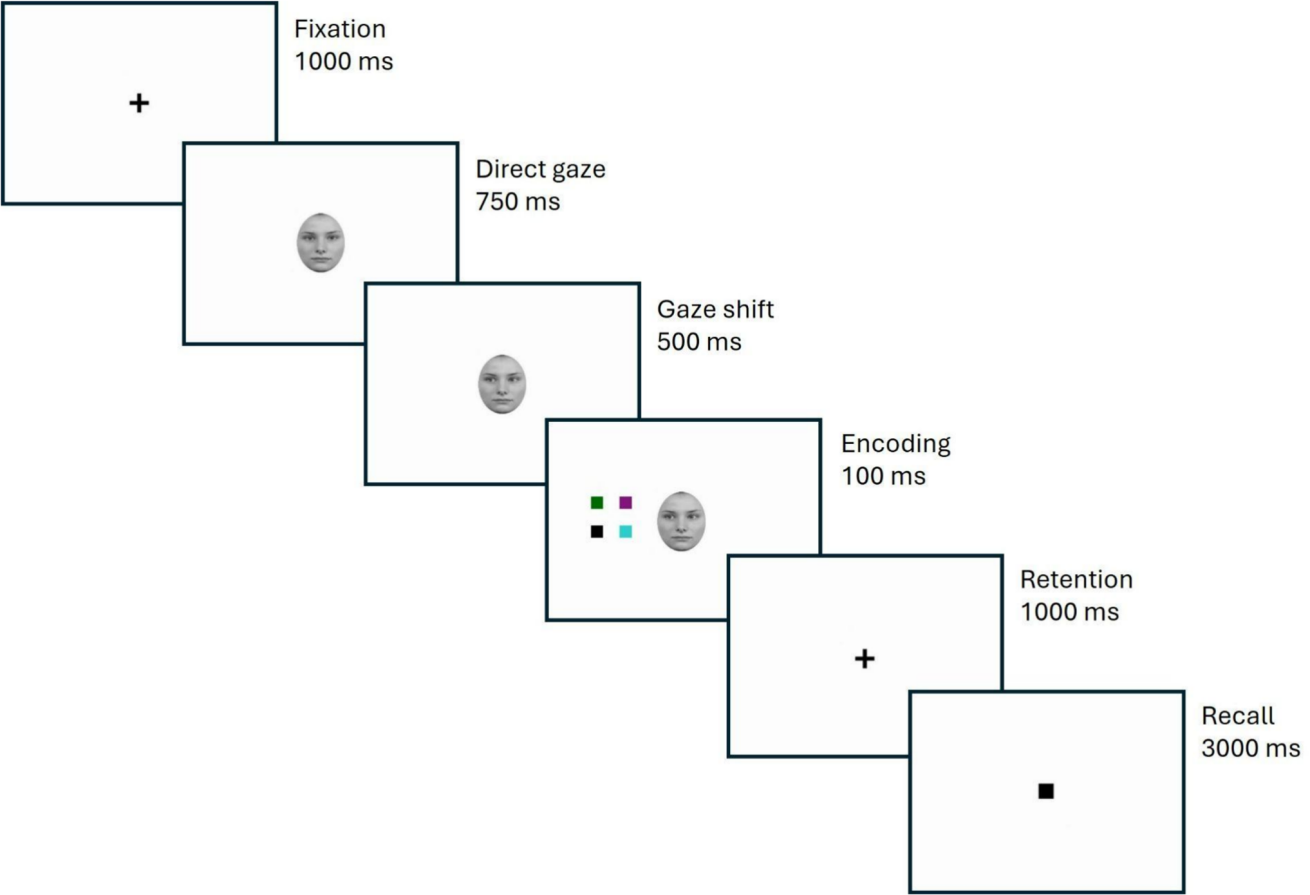
Task sequence example of a valid trial at the lowest level of load of both Experiment 1 and Experiment 2. Spatial proportions and size of stimuli in the figure are adjusted for illustrative purposes

Each trial started with a central fixation cross (1,000 ms), followed by the direct gaze version of the central cue (750 ms). The cue shifted gaze leftward or rightward after a stimulus-onset asynchrony (SOA) of 500 ms, replicating the value used in our base paradigm (Gregory & Jackson, 2017) and falling within the interval shown to reliably produce gaze-cueing effects (Frischen et al., 2007; McKay et al., 2021). Then, the encoding phase started and a set of colored squares was presented (100 ms), while the shifted gaze cue remained visible. During the retention phase (1,000 ms), only a central fixation cross was presented. In the recall phase (3,000 ms), a single colored square (the target) appeared at the center of the screen, and participants had to indicate via key press whether it had been part of the set presented during the encoding phase (see Fig. 1 for a trial sequence illustration). Feedback (“correct” or “incorrect”) followed each response. Participants were explicitly asked to indicate whether the target color presented during the recall phase had been present in the memory set shown at the beginning of each trial. The design and procedure were identical across Experiments 1 and 2. Specific details such as cue validity manipulation and instructions are described separately within each experiment’s section.

#### Data analysis

**d’**. We calculated d′ as a measure of memory sensitivity by combining Hit rates (proportion of trials in which the target was correctly detected when present) and False Alarm rates (proportion of trials in which the target was incorrectly reported as present although it was absent) for each participant, experimental block, cue validity condition, and memory load level, with the formula: $d{\prime}=z(Hits)-z(False Alarms)$. We assessed potential outliers by identifying participants whose mean d′ in the load 4 condition (i.e., the lowest memory demand) deviated by more than ±2 standard deviations from the group mean. Following this analysis, no participants were excluded from Experiment 1 or Experiment 2. To account for extreme values of Hit and False Alarm rates, we applied a standard correction: values of 0 were replaced with 1/(2N), and values of 1 were replaced with 1 − 1/(2N), where N is the number of trials on which the proportion is based (Macmillan & Kaplan, 1985; Murdock & Ogilvie, 1968). Because the number of signal (target-present) and noise (target-absent) trials varied across cue validity conditions and experiments, both N and the maximum possible d′ varied accordingly. This variation in trial number led to different maximum d′ values: approximately 2.56 for both valid and invalid trials in Experiment 1, and 3.18 for valid trials and 1.93 for invalid trials in Experiment 2.

##### Linear Integrated Speed–Accuracy Score (LISAS)

To additionally evaluate performance while accounting for both response time and accuracy, we computed the Linear Integrated Speed–Accuracy Score (LISAS; Vandierendonck, 2017). We computed the mean response time ($\mathrm{RT}$) and the proportion of errors ($\mathrm{PE}$) for each participant across experimental blocks and memory load, and we used them to calculate LISAS for each condition $i$ (i.e., cue validity) with the following formula:

$${LISAS}_{i}= {RT}_{i}+{PE}_{i}\times \frac{{SD}_{RT}}{{SD}_{PE}}$$

where ${SD}_{RT}$ is the overall standard deviation of $\mathrm{RT}$, and ${\mathrm{SD}}_{\mathrm{PE}}$ is the overall standard deviation of $\mathrm{PE}$. Therefore, lower LISAS values reflect better performance (faster and/or more accurate), whereas higher values reflect worse performance.

To examine the effects of cue validity and WM load on both d’ and LISAS, separate linear mixed-effects models (LMMs) were fitted for each experiment (Experiment 1 and Experiment 2). Each model included fixed effects for cue validity (valid, invalid), memory load (four, six, eight items), and their interaction. A random intercept for each subject was included to account for individual variability. The reference level was set to “valid” for *cue_validity* and “4” for *load*, such that model coefficients were interpreted relative to these baseline conditions. In Experiment 2, N (see description above) was included as weight to reflect reliability of d′ estimates. Statistical significance of fixed effects was assessed via Type III F-tests using Satterthwaite’s approximation for degrees of freedom, computed performing an ANOVA. Post hoc pairwise comparisons between memory-load conditions were conducted using estimated marginal means with Bonferroni correction. In the *Results* section, we report F-statistics, degrees of freedom, and *p*-values for fixed effects from the ANOVA, and model estimates (β), standard errors, *t*-values, and associated *p*-values for individual coefficients of the models where relevant. Statistical significance was evaluated at an alpha level of *p* <.05. All analyses were run using the R programme (R version 4.4.3).

## Experiment 1

### Design and procedure

Each cue validity level (valid or invalid) occurred equally within a block (50% of trials), rendering the central cue non-predictive of color stimuli location. Participants were not explicitly instructed to look at the central cue and were informed that the cue direction was unrelated to the upcoming stimuli location.

## Results

**Fig. 2:**
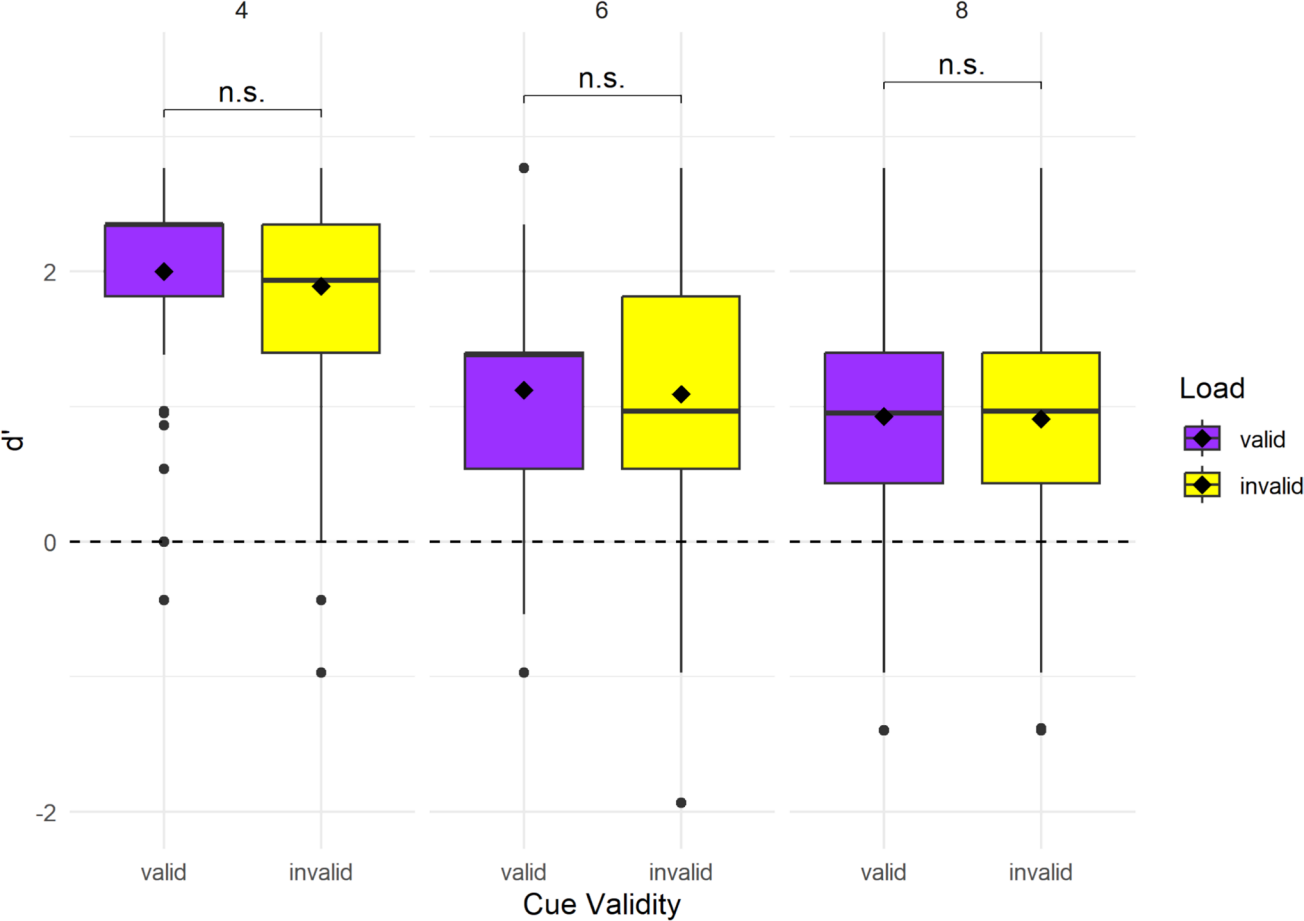
Distributions of d’ in the recollection of target stimulus of Experiment 1. Boxplots depict d’ for the valid (purple) and invalid (yellow) cue conditions for each load level (4: left boxplots, 6: central boxplots, and 8: right boxplots). The horizontal line within each box indicates the median, and the black diamond represents the mean for each cue validity and load level. The horizontal dashed line at d’ = 0 represents chance-level performance (no memory sensitivity), and values below this line indicate worse-than-chance level performance (more False Alarms than Hits). Non-significant interactions between valid and invalid cue conditions within each load level are indicated by “n.s.” Note that overall significant differences in d′ across load levels are not depicted in the figure

**d’**. The ANOVA performed on the LMM revealed a significant main effect of load, *F*(2, 1033) = 224.38, *p* <.001, indicating that perceptual sensitivity declined as memory load increased. There was no significant main effect of cue validity, *F*(1, 1033) = 1.60, *p* =.207, and the interaction between cue validity and load was also not significant, *F*(2, 1033) = 0.45, *p* =.641 (see Fig. 2). The estimated effect of cue validity at the reference level (i.e., valid and load 4) was small and non-significant (β = –0.11, *SE* = 0.073, *t*(1033) = –1.50, *p* =.135), suggesting that valid cues did not significantly enhance signal detection performance at this load level. Higher loads resulted in significantly lower d′ values: load 6 (*β* = –0.88, *p* <.001), and load 8 (*β* = –1.08, *p* <.001). The interaction terms between cue validity and load were positive but not significant (load 6: *β* = 0.08, *p* =.451; load 8: *β* = 0.09, *p* =.385), suggesting that the negative effect of invalid cues on d′ did not vary meaningfully across load levels. Post hoc comparisons confirmed that d′ was significantly higher at load 4 compared to load 6 (*p* <.001) and load 8 (*p* <.001), and higher at load 6 than load 8 (*p* <.001). Mean and SD values of d′ for each load level and cue-validity condition of Experiment 1 are reported in Table 1.

**Table 1:** Mean and SD of d’ for each load level and cue validity condition of Experiment 1

Load	Cue validity	Mean d’	SD d’
4	Valid	2.002	0.670
4	Invalid	1.892	0.715
6	Valid	1.123	0.738
6	Invalid	1.091	0.794
8	Valid	0.926	0.816
8	Invalid	0.907	0.818

**LISAS.** The ANOVA applied to the model revealed a significant main effect of load, *F*(2, 145) = 243.62, *p* <.001, indicating that LISAS scores increased with memory load, reflecting reduced performance at higher loads. The LMM estimates showed that both load 6 (β = 0.27, *SE* = 0.023, *t*(145) = 12.07, *p* <.001) and load 8 (β = 0.34, *SE* = 0.023, *t*(145) = 15.09, *p* <.001) were associated with significantly higher LISAS values compared to load 4. The main effect of cue validity was not significant, *F*(1, 145) = 0.81, *p* =.369, suggesting no overall performance benefit for valid versus invalid cues. Likewise, the interaction between cue validity and load was not significant, *F*(2, 145) = 0.14, *p* =.870, indicating that the effect of cue validity did not vary across memory load levels.

## Discussion

Memory load had a robust and significant effect on both d’ and LISAS scores: performance decreased systematically as load level (i.e., the number of items to be remembered) increased. In contrast, cue validity had no significant effect on d’ and LISAS. Furthermore, no interaction between cue validity and load was observed, indicating that the lack of the effect of cue validity was consistent across all memory load levels. These results suggest that cue validity did not facilitate WM performance in this experiment where the cue was non-predictive of stimuli location (50% validity). As participants were not explicitly instructed to attend to the central cue and were informed that its direction was unrelated to the upcoming memory stimuli, they may have deliberately avoided attending the cue. Indeed, some participants informally reported that they found the cue distracting and attempted to ignore it during the task. This suggests that top-down suppression mechanisms might have allowed for inhibiting any bottom-up orienting of attention in response to the directional gaze cue. This, in turn, prevented attentional influences on WM performance.

## Experiment 2

### Design and procedure

In Experiment 2, cue validity was set to 75%. As a result, the central cue was predictive of the color stimuli location in most cases. Participants were again not instructed to attend to the central cue but were explicitly informed that the cue direction would often indicate where the memory array would appear. This likely increased the probability that they would engage in voluntary attentional orienting toward the cued side.

## Results

**Fig. 3:**
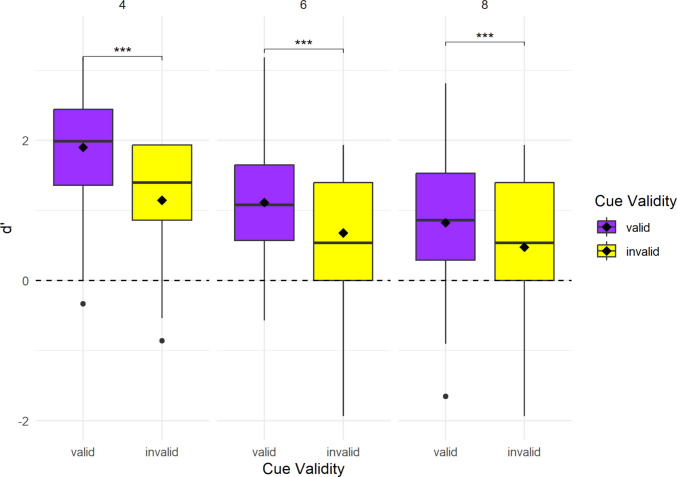
Distributions of d’ in the recollection of target stimulus of Experiment 2. Boxplots depict d’ for the valid (purple) and invalid (yellow) cue conditions for each load level (4: left boxplots, 6: central boxplots, and 8: right boxplots). The horizontal line within each box indicates the median, and the black diamond represents the mean for each cue validity and load level. The horizontal dashed line at d’ = 0 represents chance-level performance (no memory sensitivity), and values below this line indicate worse-than-chance level performance (more False Alarms than Hits). Asterisks indicate significant differences between valid and invalid cue conditions within each load level (***p <.001). Note that overall significant differences in d′ across load levels are not depicted in the figure

**d’.** The ANOVA performed on the LMM revealed significant main effects of cue validity, *F*(1, 1039) = 128.11, *p* <.001, and memory load, *F*(2, 1039) = 131.77, *p* <.001, as well as a significant interaction between cue validity and load, *F*(2, 1039) = 7.47, *p* <.001 (see Fig. 3). The estimated effect of cue validity at the reference level (i.e., valid and load 4) was high (*β* = –0.75, *SE* = 0.078, *t* (1039) = –9.62, *p* <.001). Increasing load overall reduced performance: d′ declined at load 6 (*β* = –0.79, *SE* = 0.078, *t*(1039) = −10.04, *p* <.001) and at load 8 (*β* = –1.08, *SE* = 0.078, *t*(1039) = −13.72, *p* <.001). The interaction terms showed that the cueing effect was attenuated at higher loads, as reflected by positive interaction estimates at load 6 (*β* = 0.32, *SE* = 0.111, t(1039) = 2.89, *p* =.008) and at load 8 (*β* = 0.41, *SE* = 0. 111, t(1039) = 2.67, *p* <.001). Post hoc comparisons of cue validity within each load level confirmed that valid cues significantly enhanced d′ across all loads, though the size of the benefit diminished: load 4 (*estimate* = 0.75, *p* <.001), load 6 (*estimate* = 0.43, *p* <.001), and load 8 (*estimate* = 0.35, *p* <.001) Mean and SD values of d′ for each load level and cue-validity condition of Experiment 2 are reported in Table 2.

**Table 2:** Mean and SD of d’ for each load level and cue validity condition of Experiment 2

Load	Cue validity	Mean d’	SD d’
4	Valid	1.901	0.859
4	Invalid	1.146	0.742
6	Valid	1.114	0.843
6	Invalid	0.680	0.848
8	Valid	0.825	0.807
8	Invalid	0.477	0.911

**LISAS.** The ANOVA performed on the LMM revealed significant main effects of cue validity, *F*(1, 145) = 5.81, *p* =.017, indicating overall better performance in the valid cue condition compared to the invalid one. There was also a significant main effect of load, *F*(2, 145) = 105.46, *p* <.001, with higher LISAS values observed at higher memory loads, reflecting decreased performance as memory demand increased. Model estimates showed that LISAS increased significantly at load 6 (β = 0.23, *SE* = 0.032, *t*(145) = 7.17, *p* <.001) and load 8 (β = 0.31, *SE* = 0.032, *t*(145) = 9.77, *p* <.001) relative to load 4. The interaction between cue validity and load was not significant, F(2, 145) = 0.04, *p* =.962, suggesting that the effect of cue validity on performance did not vary across load levels.

## Discussion

These results suggest that increasing cue validity (75%) and explicitly instructing that central cue direction would indicate the location of stimuli most of the time, may have enhanced participants’ use of the gaze cue, as evidenced by a statistically significant main effect of cue validity. Moreover, the interaction between cue validity and memory load suggests that participants were able to use the cue more effectively at lower loads, with the benefit of valid cues on d′ diminishing (albeit still showing its effect) as load increased. The attenuation of the cueing effect at higher loads suggests that JA presumably enhances encoding into WM depending on the available cognitive resources. The results of the speed-accuracy score showed a significant effect of cue validity on LISAS, confirming that valid cues were associated with more efficient performance overall. In summary, the enhanced cueing effect under 75% validity likely reflects a stronger engagement of top-down mechanisms, whereby participants allocated attention in accordance with task relevance.

## General discussion

The present study aimed to determine whether a strong top-down component is needed for gaze cueing to facilitate visual WM performance. Across two experiments, we manipulated the degree to which participants were encouraged to use the directional gaze, and orient their attention to the side in which a memory array would appear.

In Experiment 1, in which validity was set to 50% and participants were instructed that the direction of the cue would not be informative with respect to memory array location, we found that cue validity did not significantly modulate memory sensitivity (d’) ‒ nor the speed-accuracy score (LISAS) ‒ contrary to earlier studies reporting that gaze direction can enhance WM performance even with non-predictive cues (Gregory & Jackson, 2017; Nie et al., 2018). This absence of a validity effect, consistent across memory load levels, suggests that gaze cues ‒ when presented with 50% validity and described as non-informative ‒ may fail to automatically engage social attention mechanisms that would sufficiently influence WM encoding. One possible explanation is that participants, informed of the non-predictive nature of the cues and given no task incentive to use them, may have actively suppressed attention to the central face. This interpretation is supported by informal reports that some participants found the gaze cues distracting and chose to ignore them. This is in line with a growing body of research that has shown that gaze cueing is not purely reflexive, but is susceptible to top-down modulation depending on task relevance and social context (Dalmaso et al., 2020; Frischen et al., 2007; Wiese et al. 2014). When a gaze cue is perceived as lacking intentionality or utility, its ability to orient attention, and thus influence higher cognitive processes such as memory encoding, may be reduced (Gregory & Jackson, 2019). Thus, our findings align with theoretical perspectives positing that attentional orienting from social cues is not purely reflexive but a flexible mechanism, guided by observers’ goals, beliefs, and task context, rather than being driven by the mere presence of a social signal (Capozzi & Ristic, 2018; Zhang et al., 2023). However, another possibility is that, unlike in Gregory and Jackson (2017), our design did not include neutral (no-shift) trials, which may have reduced the salience of gaze shifts and thereby did not activate the reflexive component of attention orienting. This alternative explanation for the lack of effect in Experiment 1 could be tested in future research.

By contrast, Experiment 2 demonstrated that increasing the cue validity to 75% and explicitly informing participants about the possible cue’s usefulness significantly improved memory sensitivity and the speed-accuracy performance for valid versus invalid cues. This effect suggests that participants did integrate the predictive value of gaze cues into their cognitive strategy to solve the task. The result supports models of top-down attention in WM where reliable cues allow for prioritization of relevant spatial locations, thus facilitating encoding (Zheng et al., 2024). Awh et al. (2006) proposed that attention serves as a gatekeeper for WM, determining which information is selected and maintained. Our findings are consistent with this framework: when gaze cues were made task-relevant, they presumably enabled more effective attentional filtering and prioritization. Zheng and colleagues (2024) demonstrated that when cue informativeness is high, participants are better able to suppress task-irrelevant but visually salient items, reflecting enhanced top-down attentional control. Similarly, in our study, increasing gaze cue informativeness appeared to trigger more effective attentional prioritization, suggesting that the social cue was treated as goal-relevant when it offered strategic utility. Importantly, this effect emerged even though participants were not explicitly instructed to maintain fixation on the central gaze cue, indicating that predictive value alone can enhance the likelihood that gaze cues are treated as socially meaningful or task-relevant.

Interestingly, as cognitive load increased, the limited capacity of WM slightly constrained the extent to which this strategic orienting could translate into improved memory sensitivity in Experiment 2. This highlights the interplay between cue reliability, attentional control, and WM demands in determining the efficacy of gaze cues. In fact, we also found that WM performance declined with increasing memory load in both Experiment 1 and Experiment 2, confirming the well-established capacity limits of visual WM (Luck & Vogel, 2013) and aligning with findings that gaze following is constrained by cognitive resources, as higher WM demands are known to attenuate gaze-cueing effects (Bobak & Langton, 2015). Consistent with the memory sensitivity (d′) results, the speed-accuracy score (LISAS) was significantly lower for valid compared to invalid cues, indicating more efficient performance when the gaze cue was predictive in Experiment 2. Nevertheless, this effect did not interact with load, suggesting that the performance benefit of valid cues was stable across memory demands when cues were task-relevant.

Our findings clarify that JA is more than just reflexive gaze following. When gaze cues were non-predictive (Experiment 1), observers did not engage with them in a way that supported memory. However, when the cues were made useful (Experiment 2), they were transformed into meaningful social signals that actively enhanced WM performance. Taken together, our findings contribute to the growing literature showing that the impact of gaze cues is strongly modulated by top-down factors. At the 500-ms SOA used in both experiments, an interval where both reflexive and voluntary orienting can occur (Friesen et al., 2004), we demonstrate that a cue’s perceived utility is critical in shaping whether an observer engages in JA deeply enough to strategically support WM.

## Limitations and future directions

One possible limitation of the present study is that it manipulated cue informativeness and task relevance via instruction and probability but did not directly measure participants’ overt attention towards the cue by monitoring eye movements. Nevertheless, although eye movements can influence gaze-cueing effects, prior work shows they occur infrequently and the effect primarily reflects covert attention (McCrackin et al., 2019). Nevertheless, future studies could include eye tracking to assess whether gaze following occurred overtly, especially in the low-validity condition where we assume (but do not verify) that participants may have actively ignored the cue. Another limitation concerns the binary manipulation of cue validity (50% vs. 75%). While these values reflect standard practices in gaze-cueing paradigms, they may not capture the full continuum of how cue predictiveness affects strategic attention deployment. Additionally, although our design implicates top-down processes in modulating the influence of gaze cues on WM, we did not directly assess the role of perspective-taking or beliefs about the cueing agent. Incorporating manipulations of cue perspective could help disentangle the relative contributions of perceptual, social-cognitive, and strategic factors.

## Data Availability

The data for both experiments are available in the ZENODO public repository at the following link 10.5281/zenodo.16421651.

## Code availability

The code used to analyze both experiments are available in the ZENODO public repository at the following link 10.5281/zenodo.16421651.

## References

[CR1] Awh, E., Vogel, E. K., & Oh, S. H. (2006). Interactions between attention and working memory. *Neuroscience,**139*(1), 201–208. 10.1016/j.neuroscience.2005.08.023 16324792 10.1016/j.neuroscience.2005.08.023

[CR2] Bobak, A. K., & Langton, S. R. (2015). Working memory load disrupts gaze-cued orienting of attention. *Frontiers in Psychology,**6*, Article 1258. 10.3389/fpsyg.2015.01258 26379587 10.3389/fpsyg.2015.01258 PMC4547003

[CR3] Capozzi, F., & Ristic, J. (2018). How attention gates social interactions. *Annals of the New York Academy of Sciences,**1426*(1), 179–198. 10.1111/nyas.13854 10.1111/nyas.13854 29799619

[CR4] Cole, G. G., Smith, D. T., & Atkinson, M. A. (2015). Mental state attribution and the gaze cueing effect. *Attention, Perception, & Psychophysics,**77*(4), 1105–1115. 10.3758/s13414-014-0780-6 10.3758/s13414-014-0780-6 25737252

[CR5] Dalmaso, M., Castelli, L., & Galfano, G. (2020). Social modulators of gaze-mediated orienting of attention: A review. *Psychonomic Bulletin & Review,**27*(5), 833–855. 10.3758/s13423-020-01730-x 32291650 10.3758/s13423-020-01730-x

[CR6] Driver, J., Davis, G., Ricciardelli, P., Kidd, P., Maxwell, E., & Baron-Cohen, S. (1999). Gaze perception triggers reflexive visuospatial orienting. *Visual Cognition,**6*(5), 509–540. 10.1080/135062899394920

[CR7] Dodd, M. D., Weiss, N., McDonnell, G. P., Sarwal, A., & Kingstone, A. (2012). Gaze cues influence memory… but not for long. *Acta Psychologica,**141*(2), 270–275. 10.1016/j.actpsy.2012.06.003 22742661 10.1016/j.actpsy.2012.06.003

[CR8] Emery, N. J. (2000). The eyes have it: The neuroethology, function and evolution of social gaze. *Neuroscience and Biobehavioral Reviews,**24*(6), 581–604. 10.1016/s0149-7634(00)00025-7 10940436 10.1016/s0149-7634(00)00025-7

[CR9] Faul, F., Erdfelder, E., Lang, A.-G., & Buchner, A. (2007). G*power 3: A flexible statistical power analysis program for the social, behavioral, and biomedical sciences. *Behavior Research Methods,**39*, 175–191. 10.3758/bf03193146 17695343 10.3758/bf03193146

[CR10] Friesen, C. K., & Kingstone, A. (1998). The eyes have it! Reflexive orienting is triggered by nonpredictive gaze. *Psychonomic Bulletin & Review,**5*(3), 490–495. 10.3758/BF03208827

[CR11] Friesen, C. K., Ristic, J., & Kingstone, A. (2004). Attentional effects of counterpredictive gaze and arrow cues. *Journal of Experimental Psychology: Human Perception and Performance,**30*(2), 319–329. 10.1037/0096-1523.30.2.319 15053691 10.1037/0096-1523.30.2.319

[CR12] Frischen, A., Bayliss, A. P., & Tipper, S. P. (2007). Gaze cueing of attention: Visual attention, social cognition, and individual differences. *Psychological Bulletin,**133*(4), 694–724. 10.1037/0033-2909.133.4.694 17592962 10.1037/0033-2909.133.4.694 PMC1950440

[CR13] Gregory, S. E., & Jackson, M. C. (2017). Joint attention enhances visual working memory. *Journal of Experimental Psychology: Learning, Memory, and Cognition,**43*(2), 237–245. 10.1037/xlm0000294 27359225 10.1037/xlm0000294

[CR14] Gregory, S. E., & Jackson, M. C. (2019). Barriers block the effect of joint attention on working memory: Perspective taking matters. *Journal of Experimental Psychology: Learning, Memory, and Cognition,**45*(5), 795–808. 10.1037/xlm0000622 30024257 10.1037/xlm0000622

[CR15] Gregory, S. E., Wang, H., & Kessler, K. (2022). EEG alpha and theta signatures of socially and non-socially cued working memory in virtual reality. *Social Cognitive and Affective Neuroscience,**17*(6), 531–540. 10.1093/scan/nsab123 34894148 10.1093/scan/nsab123 PMC9164206

[CR16] Luck, S. J., & Vogel, E. K. (2013). Visual working memory capacity: From psychophysics and neurobiology to individual differences. *Trends in Cognitive Sciences,**17*(8), 391–400. 10.1016/j.tics.2013.06.006 23850263 10.1016/j.tics.2013.06.006 PMC3729738

[CR17] Macmillan, N. A., & Kaplan, H. L. (1985). Detection theory analysis of group data: Estimating sensitivity from average hit and false-alarm rates. *Psychological Bulletin,**98*(1), 185–199. 4034817

[CR18] McCrackin, S. D., Soomal, S. K., Patel, P., & Itier, R. J. (2019). Spontaneous eye-movements in neutral and emotional gaze-cuing: An eye-tracking investigation. *Heliyon*. 10.1016/j.heliyon.2019.e01583 31183437 10.1016/j.heliyon.2019.e01583 PMC6497925

[CR19] McKay, K. T., Grainger, S. A., Coundouris, S. P., Skorich, D. P., Phillips, L. H., & Henry, J. D. (2021). Visual attentional orienting by eye gaze: A meta-analytic review of the gaze-cueing effect. *Psychological Bulletin,**147*(12), 1269–1298. 10.1037/bul0000353 35404635 10.1037/bul0000353

[CR20] Mundy, P. (2018). A review of joint attention and social-cognitive brain systems in typical development and autism spectrum disorder. *European Journal of Neuroscience,**47*(6), 497–514. 10.1111/ejn.13720 28922520 10.1111/ejn.13720

[CR21] Murdock, B. B., Jr., & Ogilvie, J. C. (1968). Binomial variability in short-term memory. *Psychological Bulletin,**70*(4), 256–262. 10.1037/h0026259 5722567 10.1037/h0026259

[CR22] Nie, Q. Y., Ding, X., Chen, J., & Conci, M. (2018). Social attention directs working memory maintenance. *Cognition,**171*, 85–94. 10.1016/j.cognition.2017.10.025 29121587 10.1016/j.cognition.2017.10.025

[CR23] Nuku, P., & Bekkering, H. (2008). Joint attention: Inferring what others perceive (and don’t perceive). *Consciousness and Cognition,**17*(1), 339–349. 10.1016/j.concog.2007.06.014 17964811 10.1016/j.concog.2007.06.014

[CR24] Peirce, J. W., Gray, J. R., Simpson, S., MacAskill, M. R., Höchenberger, R., Sogo, H., Kastman, E., & Lindeløv, J. (2019). Psychopy2: Experiments in behavior made easy. *Behavior Research Methods,**51*(1), 195–203. 10.3758/s13428-018-01193-y 30734206 10.3758/s13428-018-01193-y PMC6420413

[CR25] R Core Team. (2025). *R: A language and environment for statistical computing*. R Foundation for Statistical Computing.

[CR26] Teufel, C., Alexis, D. M., Clayton, N. S., & Davis, G. (2010). Mental-state attribution drives rapid, reflexive gaze following. *Attention, Perception, & Psychophysics,**72*(3), 695–705. 10.3758/APP.72.3.695 10.3758/APP.72.3.695 20348576

[CR27] Vandierendonck, A. (2017). A comparison of methods to combine speed and accuracy measures of performance: A rejoinder on the binning procedure. *Behavior Research Methods,**49*(2), 653–673. 10.3758/s13428-016-0721-5 26944576 10.3758/s13428-016-0721-5

[CR28] Wiese, E., Wykowska, A., Zwickel, J., & Müller, H. J. (2012). I see what you mean: How attentional selection is shaped by ascribing intentions to others. *PLoS One,**7*(9), Article e45391. 10.1371/journal.pone.0045391 23049794 10.1371/journal.pone.0045391 PMC3458834

[CR29] Wiese, E., Wykowska, A., & Müller, H. J. (2014). What we observe is biased by what other people tell us: Beliefs about the reliability of gaze behavior modulate attentional orienting to gaze cues. *PLoS One,**9*(4), Article e94529. 10.1371/journal.pone.0094529 24722348 10.1371/journal.pone.0094529 PMC3983279

[CR30] Wykowska, A., Wiese, E., Prosser, A., & Müller, H. J. (2014). Beliefs about the minds of others influence how we process sensory information. *PLoS One,**9*(4), Article e94339. 10.1371/journal.pone.0094339 24714419 10.1371/journal.pone.0094339 PMC3979768

[CR31] Yin, X. (2022). Influences of eye gaze cues on memory and its mechanisms: The function and evolution of social attention. *Frontiers in Psychology,**13*, Article 1036530. 10.3389/fpsyg.2022.1036530 36312084 10.3389/fpsyg.2022.1036530 PMC9614344

[CR32] Zhang, X., Dalmaso, M., Galfano, G., & Castelli, L. (2023). Tuning social modulations of gaze cueing via contextual factors. *Psychonomic Bulletin & Review,**30*(3), 1004–1010. 10.3758/s13423-022-02211-z 36344853 10.3758/s13423-022-02211-z PMC10264287

[CR33] Zheng, W., Sun, Y., Wu, H., Sun, H., & Zhang, D. (2024). The interaction of top–down and bottom–up attention in visual working memory. *Scientific Reports,**14*(1), Article 17397. 10.1038/s41598-024-68598-y 39075215 10.1038/s41598-024-68598-y PMC11286856

